# Distinct Olfactory Cross-Modal Effects on the Human Motor System

**DOI:** 10.1371/journal.pone.0001702

**Published:** 2008-02-27

**Authors:** Simone Rossi, Alberto De Capua, Patrizio Pasqualetti, Monica Ulivelli, Luciano Fadiga, Vincenzo Falzarano, Sabina Bartalini, Stefano Passero, Daniele Nuti, Paolo M. Rossini

**Affiliations:** 1 Dipartimento di Neuroscienze, Sezione Neurologia, University of Siena, Siena, Italy; 2 Dipartimento di Neuroscienze, Sezione Psichiatria, University of Siena, Siena, Italy; 3 Medical Statistics & Information Technology, AFaR-Fatebenefratelli Association for Research, Isola Tiberina, Rome, Italy; 4 AFaR-Dipartimento di Neuroscienze, S.Giovanni Calibita, Fatebenefratelli Isola Tiberina, Rome, Italy; 5 Casa di Cura San Raffaele Cassino e IRCCS San Raffaele Pisana, Rome, Italy; 6 Dipartimento di Scienze Biomediche, Sezione di Fisiologia Umana, University of Ferrara, Ferrara, Italy; 7 Department of Orthopedics, Radiology and Otolaryngology, Istituto di Discipline Otorinolaringologiche, University of Siena, Siena, Italy; 8 Clinica Neurologica, University Campus Biomedico, Rome, Italy; 9 The Italian Institute of Technology, Genova, Italy; Harvard Medical School, United States of America

## Abstract

**Background:**

Converging evidence indicates that action observation and action-related sounds activate cross-modally the human motor system. Since olfaction, the most ancestral sense, may have behavioural consequences on human activities, we causally investigated by transcranial magnetic stimulation (TMS) whether food odour could additionally facilitate the human motor system during the observation of grasping objects with alimentary valence, and the degree of specificity of these effects.

**Methodology/Principal Findings:**

In a repeated-measure block design, carried out on 24 healthy individuals participating to three different experiments, we show that sniffing alimentary odorants immediately increases the motor potentials evoked in hand muscles by TMS of the motor cortex. This effect was odorant-specific and was absent when subjects were presented with odorants including a potentially noxious trigeminal component.

The smell-induced corticospinal facilitation of hand muscles during observation of grasping was an additive effect which superimposed to that induced by the mere observation of grasping actions for food or non-food objects. The odour-induced motor facilitation took place only in case of congruence between the sniffed odour and the observed grasped food, and specifically involved the muscle acting as prime mover for hand/fingers shaping in the observed action.

**Conclusions/Significance:**

Complex olfactory cross-modal effects on the human corticospinal system are physiologically demonstrable. They are odorant-specific and, depending on the experimental context, muscle- and action-specific as well. This finding implies potential new diagnostic and rehabilitative applications.

## Introduction

Olfaction is an ancestral sense which is essential for neocortex development [1] as well as for wild animals' survival. If the lion were unable to smell the scent of the gazelle, he would never catch it. In humans' life, there is no need to catch the gazelle, but olfaction still has some positive physiological cross-modal influence on several behavioural domains as attention [2], emotion [3], memory [4], [5], airflow motor control [6], scent tracking [7]. Moreover, when olfaction is coupled with visual inputs during grasping actions, it may favour the processing and the selection of goal-directed movements [8]. However, neurophysiological mechanisms by which olfactory stimuli can modulate the excitability of the motor system controlling hand muscles are to date still unknown. Transcranial magnetic stimulation (TMS) may offer the possibility to address this question in a fruitful and innovative way.

Indeed, by applying single TMS pulses on the scalp overlying the hand motor cortex, the amplitude of motor evoked potentials (MEPs) recorded from the contralateral target muscles, reflects physiological properties of the motor system [9], [10], either during voluntary reaching and grasping actions [11] or even during motor imagery tasks [12]–[17].

By using TMS, it has been additionally demonstrated that the corticospinal (CS) system of humans observing actions is specifically facilitated as if they were internally and sub-threshold replicating what they are look at [18] and that this effect has a strict time specificity for the kinematics of the observed action [19], [20], [21]. These findings parallel experimental results in monkeys, demonstrating that same ventral premotor neurons, called mirror neurons, discharge both during the execution of an action and during the observation of a similar action performed by another individual [22]. Mirror neurons may also be cross-modally activated by auditory stimulation, as demonstrated by single neuron recordings performed in monkey premotor cortex while the animal is listening to action-related sounds (e.g. the sound of pinprick peanuts [23]). This cross-modal activation depends upon the symbolic content of the listened action (i.e, feeding) and may have important physiological and behavioural consequences [24].

Similar findings gathered in humans by functional magnetic resonance imaging (fMRI) show that a left temporo-parietal-premotor network becomes active when individuals were listening to sentences describing actions [25] or to the mere sound of an action [26]. Notably, the degree of hanger (i.e., motivation for food) seems to selectively modulate hemodynamic responses in the fronto-parietal neural network that mediates the perception of others' grasping actions toward food [27]. Activation of these premotor neurons may form the basis for motor cortex activation, as disclosed by increased motor responses from hand muscles following TMS during listening of action-related sounds [28].

**Figure 1:**
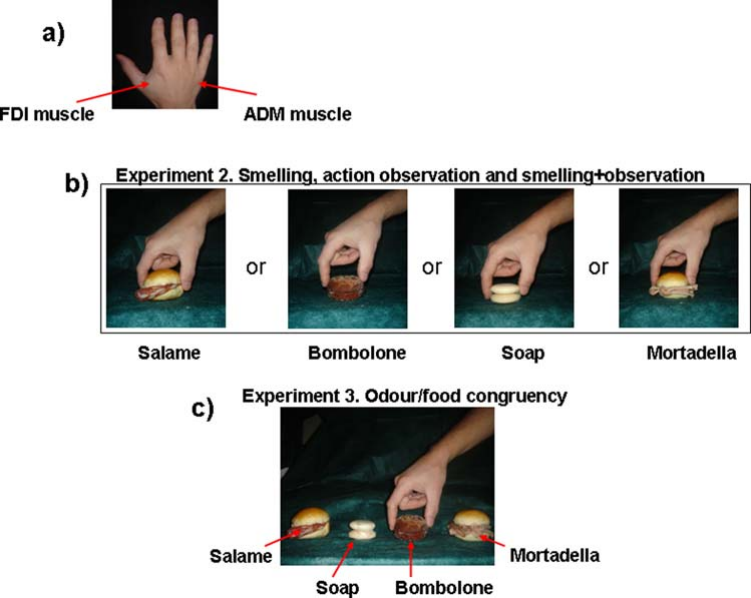
Experimental designs. Panel a): The two hand muscle from which motor evoked potentials (MEPs) were recorded. Panel b): Experiment 2. Here, the four food or non-food objects were separately presented. Within each of these main conditions, subjects were asked to observe the grasping (with or without the corresponding odour) or to simply smell the object without observing food or soap. “Salame” is the Italian name of salami, “Mortadella” represents the English term “balony”, and “Bombolone” is the Italian word to define something like a custard filled donut. Panel c): Experiment 3. Here, the four objects were simultaneously presented. The subjects smelled a single odorant (i.e.,*bombolone*), while observing the experimenter reaching and grasping three edible objects (one of which was *bombolone*) or a non-edible object of a similar shape (soap).

The symbolic content of the observed or listened action seems therefore pivotal for the cross-modal modulation of the activity of premotor and motor areas, both in monkeys and humans. Since olfaction is the most ancestral sense, moreover encompassing strong evocative components [29], we predicted that also olfactory stimuli, besides the motivation induced by food vision [27], could, in parallel with action-observation, influence the excitability of the human CS system. We aimed to causally verify this novel hypothesis by using TMS to specifically quantify the degree of CS facilitation of hand muscles during action-viewing and congruent/incongruent olfactory stimulation (see Fig. 1).

## Results

### Experiment 1. Sniffing synthetic and natural odorants

**Figure 2:**
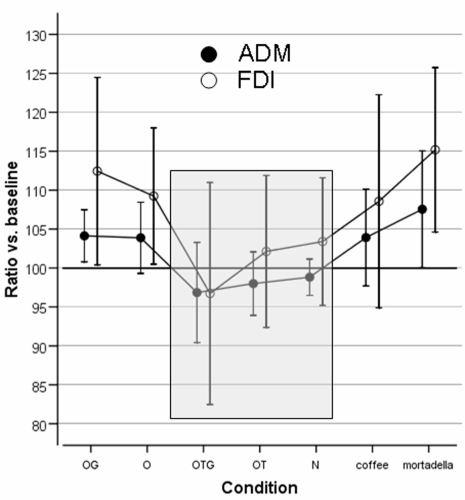
Results of Experiment 1. Mean percent changes of log-transformed MEP amplitude from hand muscles (FDI and ADM) versus the control condition Basal (the 100% reference line). The condition “Neutral” (N) was similar to Basal. Sniffing unimodal olfactory (O), bimodal olfacto-gustative (OG), and natural odorants as *coffee* and *mortadella* increased MEPs' size. Such facilitation of corticospinal output disappeared when sniffing odorants with a trigeminal component (OT and OTG, grey area). Notably, no cognitive tasks except sniffing are required to subjects. Statistics are in the text.

The first question we addressed was whether olfaction exerts some general effects on CS excitability. To this purpose we measured the excitability of the CS system driving two different hand muscles (*First Dorsal Interosseous* (FDI) and *Abductor Digiti Minimi*, ADM) during sniffing of five synthetic and two natural odorants (*Coffee* and “*Mortadella”,* a typical Italian pork-derived salami). Synthetic odorants were characterized by different combinations of olfactory (O), gustatory (G) and trigeminal (T) components. An odourless stimulation condition (“*Neutral”*) was included as control. TMS-induced motor evoked motor potentials (MEPs) simultaneously recorded from the two hand muscles were analyzed by ANOVA (see Methods). Ratios vs. baseline of MEP amplitude values collapsing the seven experimental conditions (OG, O, OTG, OT, N, “*Coffee”*, “*Mortadella”*) were similar for the two muscles [main factor MUSCLE: F(1,9) = 1.204, p = .301, eta-squared = 12%]. Conversely, MEP amplitude ratios significantly changed across conditions [F(6,54) = 8.474; p<.001], with about a half of MEP-ratios variance accounted for by such factor (eta-squared = 49%). This demonstrates that the excitability of the CS system was differentially modulated by the presented odours. However, no differences were found for the two muscles as shown by the lack of significance of the interaction MUSCLE*CONDITION [F(6,54) = 0.655, p = 0.686, eta-squared = 7%] (Fig. 2). Comparisons between conditions (Tukey's HSD method) indicated two different homogeneous subsets: the first included the *Neutral* stimulus as well as the two sniffing conditions which included a trigeminal component in the odorant (OT, OTG). Differences within this subset were not significant (p =  0.555), and the 95% confidence intervals (CI) for each of these three conditions always contained the 100% reference line, indicating a non-significant variation with respect to baseline MEP (Fig. 2). The second subset included unimodal olfactory (O), bimodal olfactory-gustative (OG) synthetic odorants as well as the two natural odorant “*Coffee*” and “*Mortadella*”: in this case, differences within this subset resulted in an overall p-value of 0.349. The 95% CI for these conditions did not cross the 100% reference line (only “*Coffee”* was not completely above this line), thus indicating a consistent and homogeneous increase of MEPs size. Summarizing, this Experiment demonstrates that the excitability of the motor system is modulated by olfaction. This modulation is specifically dependent upon the characteristics of the sniffed smell, being maximal with olfactory-gustative stimuli and absent with trigeminal ones.

### Experiment 2. Smell, observation of grasping actions, and smelling during observation

After demonstrating that olfaction *per se* may exert some effects on CS excitability and that OG stimuli were the most effective in facilitating the motor system, we investigated the presence of cross-modal interactions between olfaction and observation of grasping actions. A factorial design was used for this purpose, with Observation (two levels: no-Obs and Obs) and Smell (two levels: no-Smell and Smell) as the main two within-subjects factors. So, the following four experimental conditions were obtained for each object: a) no-Obs, no-Smell (baseline); b) no-Obs, Smell; c) Obs, no-Smell; d) Obs, Smell. Subjects were requested to *smell* and/or *observe the experimenter while grasping* three foods (*“sandwich with Mortadella”*, *“sandwich with Salame”* and *“Bombolone”*, see Methods, and one non-food-related, but still carrying a pleasant odour, object, “*Soap*”). To be noted that, differently from experiment 3, the combination of observation and smell was always congruent, i.e. observing grasping while smelling the odour of the to-be-grasped object (Fig. 1).

Since *Mortadella*, the natural odorant that clearly increased MEP amplitude in Experiment 1, was one of the four different objects used in this experiment, data from the current “Smell” condition can be also used to confirm the reliability of one of the findings of the previous experiment. Indeed, the magnitude of MEP increase (about 12% versus *Basal*, after logarithmic transformation) during Smell was similar to that of Experiment 1.

**Figure 3:**
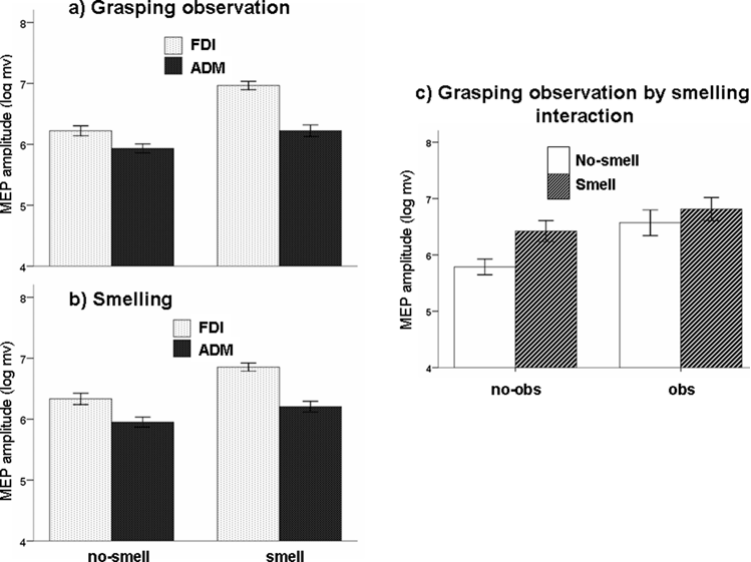
Results of Experiment 2 (only food objects). Graphical representations of the main significant interactions: a) an higher MEP increase occurred in FDI than in ADM when the subjects observed a grasping movement; b) similarly, an higher MEP increase occurred in FDI than in ADM when the subjects smelled a food; c) even if “smelling” produces a lower MEP increase with, than without, concomitant grasping observation, a significant further facilitation (27% in the original µV scale) was found.

Table 1 reports the details of factorial ANOVA. SMELL, as main effect, produced a log-MEP increase of about 7% (p<.001); estimated marginal means (in the original scale of raw data) indicated that no-smell average MEP size was 482 µV and smell-MEP was 748 µV, corresponding to a 55% increase. A quite similar 10% increase of log-MEP was attributable to the main effect of Obs (p<.001); estimated marginal means (in the original scale) indicated that MEP increased from 448 to 806 µV, corresponding to a 80% increase. The significant double interaction MUSCLE*OBS (p = .007) and MUSCLE*SMELL (p = .002) respectively indicated that observation and smelling increased FDI-MEP more than ADM-MEP (13% vs. 6% and 9% vs. 5%, respectively; Fig 3a and 3b). Less relevantly, the observation of grasping a *Bombolone* increased MEP more than the observation of grasping a sandwich with *Mortadella* or *Salame*, resulting in an OBJECT*OBS double interaction (p = .013). Even the significant triple interaction MUSCLE*OBJECT*SMELL was not much relevant (simply indicating that the MUSCLE*SMELL interaction –mentioned above- was quite different for the three objects, since smelling *Mortadella* produced a parallel MEP increase in both muscles, while smelling *Salame* and *Bombolone* produced a differential pattern with higher increase in FDI muscle) [graphical data for each food object not shown].

**Table 1:** Main analysis (factorial ANOVA) of the Experiment 2 for the three foods

Factors	F	Greenhouse-Geisser d.f.	p-value	Partial Eta-Squared
MUSCLE	6.971	1,9	0.027	0.44
OBJECT (Foods)	2.960	2,17.8	0.078	0.25
OBS	31.836	1,9	0.000	0.78
SMELL	47.583	1,9	0.000	0.84
MUSCLE * OBJECT	3.410	1.4,12.8	0.077	0.27
MUSCLE * OBS	11.949	1,9	0.007	0.57
OBJECT * OBS	5.636	2,17.6	0.013	0.39
MUSCLE * OBJECT * OBS	3.364	1.5,13.3	0.077	0.27
MUSCLE * SMELL	18.781	1,9	0.002	0.68
OBJECT * SMELL	2.379	1.5,13.7	0.138	0.21
MUSCLE * OBJECT * SMELL	4.544	1.8,16.4	0.029	0.34
OBS * SMELL	6.729	1,9	0.029	0.43
MUSCLE * OBS * SMELL	6.311	1,9	0.033	0.41
OBJECT * OBS * SMELL	1.926	1.3,12.1	0.191	0.18
MUSCLE * OBJECT * OBS * SMELL	0.180	1.7,14.9	0.798	0.02

Much more relevant for the study's objective is the OBS*SMELL interaction (Fig. 3c and Table I). The Smell-induced MEP increase was more evident when subjects did not observe grasping actions: indeed, Smell produced a MEP increase of about 11% without concomitant observation (89% in the original µV scale) and of about 4% (27% in the original µV scale) when associated to observation. This suggested a lack of multiplicative effect due to the association Smell+Obs. However, Smell produced a significant MEP increase not only “without observation” (Tukey's p = .002), but even “with observation” (p = .032), suggesting that a further –although smaller- increase of cortical excitability could be expected as biological effect of smelling. The negative OBS*SMELL interaction (Table I) coupled with the additive Smell-induced MEP increase during observation are not in logical contradiction: rather, they unveil the frequent occurrence of a discrepancy between the concepts of statistical interaction and biological interaction [30].

OBS*SMELL*MUSCLE interaction indicated that the pattern of the SMELL*OBS interaction was dependent by muscles (Table I). In fact, in the ADM muscle a slighter effect of both Smell and Obs was observed, without any interaction between them. As opposite, in the FDI a stronger increase of both Smell and Obs was found, their interaction being significant since the increase due to Smell during observation was again smaller than the increase due to Smell alone (i.e., without grasping observation).

**Figure 4:**
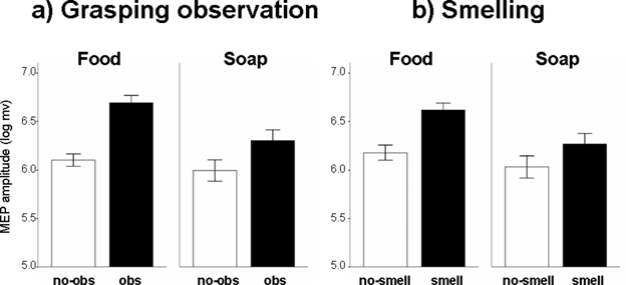
Results of Experiment 2 (Food vs Soap). Graphical representations of the main significant interactions: a) an higher MEP increase occurred when the subjects observed a grasping movement toward a food object than toward a non-food object; b) an higher MEP increase occurred when the subjects smelled a food object than a non-food object

Since the slight interactions involving OBJECT did not have a disrupting effect on lower-order terms and since type III sum-of-squares was used (allowing to adjust each source of variation for all terms), *Mortadella*, *Salame* and *Bombolone* could be collapsed by averaging, so that a unique object “Food” could be defined. The new object Food was compared to a non-alimentary object of the same shape and size (*Soap*) in a further four-way ANOVA. Here the most interesting findings were the interactions OBJECT*OBS [F(1,9) = 5.035; p = .052] and, even more, OBJECT*SMELL [F(1,9) = 8.010; p = .020]: this indicates that Smell (and Obs to a lesser extent) produced a larger MEP increase when the object was a food (Fig. 4).

Thus, this experiment, besides confirming the results of Experiment 1 (food-related odours facilitate the CS system more than non-food related ones), shows that, during grasping observation, the muscle that the observer would activate as prime-mover to perform the seen action was more facilitated than the control muscle. More importantly, it further shows that smelling the flavour of a food while observing its grasping exerts an additional and significant CS facilitation.

### Experiment 3. Congruency between odorant and observed grasped food

Here we were aiming at demonstrating the degree of specificity of the synergic effect of smelling while observing a grasping action. Indeed, the possibility remains that what we showed by Experiment 2 was the effect of two independent factors on the CS system, rather than the demonstration of a true, cross-modal interaction between olfaction and grasping. To this purpose, we asked subjects to observe the same grasping actions we presented in Experiment 2, but in this case they were always smelling the flavour of the *Bombolone*. The prediction was that, in such multi-choice context, the congruence between the observed action and the sniffed smell should have exerted a maximal and selective facilitation of CS system. A significant interaction [F(2.5, 22.7) = 4.06; p = .024] was found in the two-way ANOVA with MUSCLE (FDI and ADM) and OBJECT (*Salame*, *Bombolone*, *Mortadella*, *Soap*) as within-subject factors. As shown in Fig. 4, differences between the four objects occurred in FDI [F(3,27) = 7.178; p = .001] and did not in ADM muscle [F(3,27) = 1.239; p = .315]. It should be noted that no MEP increase was observed in ADM (each 95% CI crossed the 100% reference line), while a clear increase was found in case of full congruence between smelling *Bombolone* and observing the grasping action of the *Bombolone.* Actually, the 95% confidence interval was above the reference line even in a case of “alimentary congruency” (smelling *Bombolone* and observing the grasping action of the *Mortadella*), probably due to a particularly low standard deviation that allowed to recognise as significant also a small increase (about 5%). However, orthogonal contrasts indicated that significant differences between FDI and ADM were found comparing *Bombolone* to *Salame* (p = .031), *Mortadella* (p = .005) and *Soap* (p = .012).

## Discussion

Neuroimaging studies based on techniques measuring cerebral blood flow/metabolism (PET and fMRI) are increasingly unveiling the functional correlates of olfactory processing in humans [31], [32]. Overall, these studies have indicated that brain regional activity co-varies with odorant stimuli in a complex manner, with a distributed topography which seems dependent by the unimodal or bimodal nature of the odorant [33], [34], its degree of pleasantness [29], and memory load associated with its emotional content [4]. Depending on the weight of these factors, piriform and orbitofrontal cortex, amygdala, anterior cingulate cortex, insula, thalamus, enthorinal cortex and cerebellum may become active in odour perception and processing [32]. As a general concept, however, fMRI/PET activations are unable to provide information about the causality and chronological hierarchy of the nodes in the activated networks.

Current results extend previous neuroimaging findings, which have never shown activation of the motor cortex following odorant stimulation. This could be due to the limited temporal resolution of the techniques measuring cerebral blood flow and metabolism, which might have missed transient motor cortex signal changes in the frame of a “tonic” brain activation during smell processing, or –more probably- to the different experimental context of these studies, in which smelling was not coupled with visuo-motor tasks. Here, distinct cross-modal olfactory effects on the motor system have been observed, depending from the experimental context.

In Experiment 1, both synthetic and natural olfactory and olfacto-gustative odorants, determined a non-specific increase of CS excitability of hand muscles. This effect was not dependent upon a putative facilitation induced by the mere motor act of sniffing, since MEP amplitude changes were significant also when contrasted with the “Neutral” condition (where subjects sniffed an odourless stimulus). Moreover, the effect occurred while subjects were not engaged in specific motor, cognitive or visual tasks. Thus, a direct olfactory/motor functional link was disclosed, possibly behaviourally relevant through an unconscious preset of motor strategies automatically recruited by the smell-evoked representation of an object [8]. Notably, the effect was maximal in case of “*Mortadella*”, a traditional Italian food implying strong evocative sensations. In case of odorants including a trigeminal component, the corticospinal facilitation on hand muscles disappeared, in line with the concept that an overt noxious stimulation reduces the corticospinal output as tested by TMS [35], [36] and, more importantly, that a similar inhibitory modulation takes place during observation of painful actions [37]. Therefore, it can be hypothesized that the trigeminal component contained in the OT or OTG odorants could represent a potentially painful stimulation implying subjects' warning and unconscious avoidance reaction. Indeed, pain processing in the cranial district is mainly due to activation of the trigeminal system, and overlapping neural structures (i.e., orbitofrontal cortex, secondary somatosensory cortex, insula) [30], including the supplementary motor area [38] become active in neuroimaging studies during processing of both pain and trigeminal odorants. In humans, this mechanism may represent a behaviourally relevant remnant of wild life, in which warning and avoidance reaction toward potentially dangerous odorants are building blocks of daily survival.

Behaviourally, it has been hypothesized that, in a way analogue to that of monkey visuomotor ‘canonical’ neurons [39] which respond during object grasping and object observation, olfactory information may indeed contain both the ‘olfactory’ representation of an object and the motor representation of the most suitable hand/finger interaction with it. Increase of MEPs amplitude in the hand muscle prime mover for food-related actions during smelling food or observing+smelling food elements, and congruency between smell and the observed grasped food, provide the neurophysiological background of this mechanism in humans. Notably, the magnitude of MEP facilitation induced by smelling (Fig. 3a and b) was almost comparable to that induced by action observation, the latter being a solid result coming from previous neurophysiological studies causally addressing the role of the motor cortex in actions observation [18], [20], [28], [40]. Taken together, these results would suggest the appealing hypothesis of a cross-modal activation of the mirror-neuron system triggered by olfactory stimulation/processing, a concept that still lacks direct evidence in monkeys and humans, but plausible if one considers that: i) other sensory modalities, as auditory and visual inputs, are indeed able to activate a widespread frontoparietal system compatible with the putative mirror activity in humans [25], [26]; ii) the motivation to eat modulated hemodynamic responses in such system when hungry subjects observed grasping food [27]; iii) single-neuron recordings in monkeys showed that both the observation and hearing of food-related actions activate motor programs related to eating behaviours [41].

The key feature of the current study is that the selectivity of olfacto-motor facilitation on hand muscles was strictly dependent from the experimental context: the smell-induced modulation of corticospinal excitability was totally unspecific when observation of grasping food was not required, as demonstrated by Experiment 1. In Experiment 2, MEPs evoked in the muscle prime mover for the observed grasping (i.e., FDI muscle) were generally higher than those evoked in the ADM muscle, but in both muscles MEPs amplitude was clearly facilitated during smelling versus “*Basal*” conditions. This could represent the neurophysiological evidence of an unspecific olfactory “limbic” drive on the human corticospinal system, on which the alimentary valence of the observed grasped object might play a role to channel more specific motor commands, as suggested by the results of the last analysis of Experiment 2 (Food versus Soap, Fig. 4).

**Figure 5:**
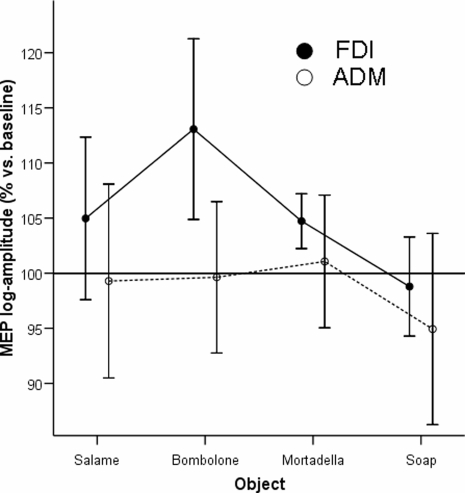
Results of Experiment 3. Mean percentual changes of MEP amplitude versus “*Basal*” in the FDI muscle, which was prime mover for the observed grasping action, and in the ADM muscle which has no functional role in grasping. Only the congruency between the odour of *bombolone* with the observation of grasping a *bombolone,* but not other food or non-food objects, produced a significant increase of MEP size, which selectively took place in the prime mover. No changes are observed in the ADM muscle. Statistics are in the text.

However, only the full congruency between the smell of *Bombolone* and the observation of grasping “*Bombolone*” (Experiment 3, Fig. 5) produced a clear-cut and specific facilitatory modulation of FDI MEPs amplitude, associated with no significant changes of ADM MEPs. “Alimentary congruency” (i.e., smelling *Bombolone* and observing the grasp of other food objects, but not of soap) had weaker, but still prime-mover specific, effects on MEPs amplitude. Thus, such strict specificity appeared only in the context of a multi-choice discriminative task, requiring an implicit matching between a given olfactory information and the activation of the motor representation required to grasp the corresponding food. A non-mutually exclusive cognitive hypothesis explaining such olfacto-motor effect could consider the “context closure” model [42], in which the congruency between smell and observed food could represent the comprehensive and congruent closure of information processing occurring when expectations are terminated. This represents a plausible explanation, independent from the internal motor repertoire. Indeed, lesion studies indicating a clear dysfunction of mirror neurons system due to ischemic stroke, haemorrhage or neoplasms are still lacking [43]. However, the context closure hypothesis does not explain MEP modulation during Experiment 1, because it appeared in absence of specific cognitive tasks.

The magnitude of smell-induced facilitation of the motor system, when coupled with observation of gestures, is generally higher than that that induced by action observation only. Therefore, future research efforts will exploit such strategy in rehabilitative settings for mildly paretic hands. Smell-induced modulation of MEP amplitude might additionally become an objective test for anosmia confirmation in simulators, and could be applied as a diagnostic test in neurological disorders, as Parkinson's disease, in which hyposmia could represent one of the earlier clinical features [44].

## Materials and Methods

Twenty-four healthy right-handed (Oldfield questionnaire score >85%) volunteers (12 males; age range 22–46 years) were enrolled in the study after the approval of the procedure by the local Ethical Committee. All gave a written informed consent to the study. Three different experiments were carried out on 10 subjects each. Four subjects (3 males) participated to more than one experiment. The subjects sat fully relaxed on an armchair, in a well ventilated room, with their right forearm resting on a pillow and their hand kept pronated, in a natural position. According to the conditions of the experimental design, they were instructed to look at a fixation point positioned in front of them, or to the experimental actions or objects presented on a uniform green background. In all cases, they could not have visual access either to the equipment display or to their hands. All subjects had their last meal the evening preceding the day of the experiment, that was carried out at lunch time. All of them were neurologically normal, not assuming neuroactive drugs and not complaining of olfactory deficits. Rhinological examination performed on all subjects was reported as normal by a rinhologist MD.

### Stimulating and recording procedures

TMS was carried out via an eight-shaped focal coil connected with a monophasic Magstim 200 stimulator. The coil, angled of about 45° from the midline with its handle pointing backwards, was positioned on the region of the left hemiscalp triggering MEPs from the contralateral examined hand muscles with the minimal threshold (hot spot), as defined according to international standards [45]. The hot spot was marked on the scalp to allow the same coil positioning during the experiments, and the TMS intensity was adjusted to produce simultaneous fairly stable basal MEPs of 400–800 µV in the right First Dorsal Interosseous (FDI) and of 200–500 µV in the Abductor Digiti Minimi (ADM) muscles. This intensity corresponded to about 110–115% of the individual motor threshold.

The choice of these two muscles (Fig. 1) was motivated by the fact that the grasping actions (see later) to be observed deeply involved the FDI muscle, which is a prime mover for pinch grasping of small objects, but not the ADM muscle, which has no crucial functional roles in pinch grasping, but shares with the FDI cortical representation, corticospinal control and peripheral innervation [14], [46].

Ag-AgCl adhesive electrodes were applied over the muscles in a belly-tendon bipolar montage, with the active electrode placed on the motor point of each muscle. A total time epoch of 200 ms was analyzed in each trial, the first 100 ms serving as pre-trigger analysis time to monitor and exclude those trials contaminated by unwanted background EMG activity. MEPs were recorded by a four-channel electromyograph (Phasis, EBNeuro), with a bandpass filter of 20Hz-5 KHz, sampled at 20 KHz, with a gain range of 0.1–1 mV. An acoustic feed-back monitoring the EMG background activity was given to the subject through a loudspeaker.

Pairs of MEPs from the two muscles were recorded in each experimental condition, each TMS pulse being spaced 10–15 seconds from the previous one. Only MEPs with the same latency and morphology were stored for post-processing: a MEP-to-MEP latency onset difference of 1 ms occurring in the FDI or ADM responses was considered as proof of activation of a different neural pool (or as warning for a slight voluntary contraction). They were thus discarded from the analysis, even in absence of evident contamination from EMGraphic activity. MEPs were also discarded if small EMG bursts (even less than 50 µV) preceded the TMS in one of the two traces without latency shifts or amplitude increment. The rejection rate was about 25%, without significant differences across experimental conditions. Five pairs of MEPs (out of the 7–8 acquired for each condition) were finally stored for further analysis in each experimental condition. This apparently low number of trials/condition was due to the fact that the effects of olfaction on corticospinal excitability were unexplored previously, so we could not predict how long these effects (if any) would have persisted (i.e., a tonic effect) or if they were present only at the beginning of the olfactory stimulation and then disappeared (i.e., a sort of phasic effect).

### Experiment 1. Sniffing synthetic and natural odorants

The experimental procedure was as follows: pairs of MEPs were recorded from FDI and ADM muscles as a control (“*Basal*”). Then, pairs of MEPs were recorded for each of seven -randomly administered- sniffing conditions, including five standardised synthetic odorants (*Sniffin sticks olfactory test*) [47], routinely used for diagnosis in hyposmic and anosmic patients, plus two natural odorants (“*Coffee*” in beans and “*Mortadella*”, a typical Italian pork-derived product to make sandwiches, or “balony”). Synthetic odorants, delivered by standard penlike odour-dispensing devices, and natural stimuli were kept by the experimenter at approximately 2 cm from nostrils. Care was taken to maintain odorants out of the subject's sight and to deliver the TMS pulse 3–5 seconds from odorant stimulation.

It is known that corticospinal tract excitability is not modulated by the natural inspiration/expiration cycle [48]; thus, subjects were practiced to sniff as softly as possible, to minimize respiratory muscular activation linked with deep inspiration that would have increased corticospinal drive, thus enhancing MEPs amplitude [48], although unspecifically. However, in a control condition (“*Neutral*”) the penlike device did not dispense any odour. The remaining synthetic odorants included a pure unimodal olfactory stimulus (coffee, “O”), two bimodal olfactory-gustative stimulus (banana, “OG”) one olfactory-trigeminal stimulus (mint, “OT”), and one olfactory-trigemino-gustative stimulus (canella, “OTG”). The duration of the experiment was about 15 minutes.

### Experiment 2. Smell, observation of grasping actions, and smelling during observation (Fig. 1)

Pairs of MEPs were recorded from FDI and ADM muscles (condition “*Basal-1*”), without giving particular instructions to subjects. Afterwards, the following block design was run, including four main randomized conditions: “*Mortadella*” (see above), “*Salame*” (another pork product with a different smell of similar intensity, or “salami”), “*Bombolone*” (a fried cake with cream inside and sugar outside, something like a custard filled donut) and “*Soap*” with gentle and peasant smell, but not of alimentary origin. Within each of these main conditions, during which a single object was shown, the subjects underwent, again in a random order, three tasks including observation of a grasping action of food or soap (with or without the corresponding odour) or to smell their odour without observing food or soap. In each of these four conditions, pairs of MEPs were recorded from both muscles. During action observation without smell, subjects wore nostril-plugs to prevent awareness of odorant stimuli.

More in detail, subjects had to observe the examiner's right hand performing -with a natural movement- the reaching-grasping of (i) a small sandwich (prepared with fresh bread) containing S*alame* or *Mortadella*; (ii) a *Bombolone* or (iii) a sandwich-shaped soap, formed by two pieces of odorant soap (without alimentary valence). The shape and the dimension of all grasped elements were nearly similar.

Again within the random design, subjects observed the same grasping acts during sniffing the three observed food objects (or soap). Subjects were also tested during the mere olfactory stimulation of the three food objects and soap, without observing grasping actions or static objects. Odours of the natural food (or soap), were administered by putting objects inside a small container positioned just below subject's nostrils, preventing the subject from sight or somatic contact with the source of odour.

The choice of these natural food stimuli was motivated by the results of the Experiment 1 and by their large-scale agreeability in the population, due to their lovely and unique flavour and fragrance.

Care was taken to deliver the TMS pulse during the grasping phase or, in trials without observation tasks, after 3–5 seconds of odorant stimulation. After all conditions were run, five additional pairs of MEPs were recorded (“*Basal-2*”) in order to verify that corticospinal excitability remained stable also in the absence of visual or olfactory stimulation. The duration of this experiment was about 30 minutes.

The use of natural grasping and food stimuli instead of standardised videos and odorants was decided to render the experimental setting as much as possible realistic and evocative. Indeed, when the experiment was over, most of subjects actually asked to eat the remaining “experimental material” (with the obvious exception of the soap).

### Behavioural measures

Before the TMS experiment, subjects were asked to rate on a 0–100 analogue-visual scale (alimentary VAS) how much they were fond of *Salame*, *Mortadella* or *Bombolone*.

### Experiment 3. Congruency between odorant and observed grasped food

Subjects were instructed to look at the scene in front of them showing simultaneously the four objects of the Experiment 2 (two sandwiches with *Salame* or *Mortadella*, a *Bombolone* and a piece of soap) aligned horizontally on a plane (Fig. 1), whose left-right order was counterbalanced. In this experiment, as in the previous ones, five pairs of MEPs were simultaneously recorded from the two hand muscles (“*Basal*”). Then, subjects had to look at the examiner's right hand performing grasping actions of one of the four objects while they were presented with the smell of the *Bombolone*. Timing of the TMS pulse, inter-trial intervals and procedures of olfactory stimulation were as in the previous experiments. Pairs of MEPs were recorded duirng each of these four conditions (“*Salame*”, “*Mortadella*”, *Bombolone*, “*Soap*”), whose order was randomized. Thus, a full congruence between odorant and grasped object took place exclusively in the condition “*Bombolone*”. It should be noted that while the two conditions “*Salame*” and “*Mortadella*” shared with the odour of *Bombolone* a sort of “alimentary congruency”, the condition “*Soap*” served as a control, since in this case an alimentary odorant was coupled with a grasping action of a non-food object.

### Data analysis

Five FDI and five ADM MEPs were averaged for each condition, and peak-to-peak maximal amplitude was calculated off-line. Latencies were not analyzed due to experimental constrains (see “stimulation and recording” paragraph). Considering that the distribution of FDI and ADM MEPs amplitude data followed approximately a log-normal pattern, we performed the log-trasformation to improve their gaussianity, to stabilise variances across conditions and to reduce outliers effects [49]. After this transformation, for Experiment 1 and Experiment 3, ratios between the averages in the different conditions and the average of baseline were computed. Although ratios are usually not suitable for linear models and should often be transformed by means of other mathematical operators (typically arcsin), for the current data-set this linearization did not produce a relevant effect on scale (variance, coefficient of variation) and shape (kurtosis, skewness). Hence, simple ratios between log-values were used here as dependent variable. Analysis of variance (ANOVA) was used as statistical procedure to reveal the significance (F and p-values) and the effect size (eta-squared) of the various sources of variations considered in the study. More in detail, in the Experiment 1, objects (seven levels) and muscles (two levels) were the two within-subjects factors in the two-way ANOVA. In the Experiment 3, objects (four levels) and muscle (two levels) were the two within-subjects in the two-way ANOVA. Post-hoc testing was carried out whenever appropriate.

Data from Experiment 2 were analysed differently, in the attempt to better fit the different objectives of this part of the study and the peculiar experimental design. Indeed, since the objective of Experiment 1 and 3 were to assess the hypothesised facilitation of MEP (eventually muscle-specific) attributable to some types of odour (Exp.1) and to the congruency between observation and smelling facilitation (Exp.3), a “facilitation measure” had to be used, as the dependent variable and the ratio between MEPs in each condition and MEP at baseline was chosen. Conversely, the Experiment 2 aimed to address the issue of the interaction between grasping observation and smelling, thus a factorial design was considered the best approach. For such a design, the ratios vs. baseline would not allow a more direct estimate of the “observation” effect, of the “smelling” effect and of their interaction, eventually modulated by muscles and type of object. Therefore, the first factorial design included Observation (two levels: no-Obs and Obs), Smell (two levels: no-Smell and Smell), Muscle (two levels: FDI, ADM), Object (three levels: *Salame*, *Mortadella*, *Bombolone*) as within-subjects factors. In the second factorial analysis of Experiment 2, the three alimentary objects were collapsed in one level (Food) and contrasted to a non-alimentary object (Soap).

Since the subjects' pleasantness towards odorants could play an interfering role, VAS scores were considered as covariates in a mixed model with “Subject” as random-effects factor and the others factors (specific for each experiment) as fixed-effects factors. Since no changes occurred in terms of effects of interest, these findings will not be reported in the Results section.
